# Use and Prescription of Direct Oral Anticoagulants in Older and Frail Patients with Atrial Fibrillation: A Multidisciplinary Consensus Document

**DOI:** 10.3390/jpm12030469

**Published:** 2022-03-15

**Authors:** Marco Proietti, Marina Camera, Maurizio Gallieni, Luigi Gianturco, Antonio Gidaro, Carlo Piemontese, Giuseppe Pizzetti, Franco Redaelli, Barbara Scimeca, Carlo Sebastiano Tadeo, Matteo Cesari, Giuseppe Bellelli, Laura Adelaide Dalla Vecchia

**Affiliations:** 1 Geriatric Unit, IRCCS Istituti Clinici Scientifici Maugeri, 20138 Milan, Italy; marco.proietti@unimi.it (M.P.); macesari@gmail.com (M.C.); 2 Department of Clinical Sciences and Community Health, University of Milan, 20122 Milan, Italy; 3 Liverpool Centre for Cardiovascular Science, University of Liverpool and Liverpool Heart & Chest Hospital, Liverpool L7 3FA, UK; 4 Centro Cardiologico Monzino IRCCS, 20138 Milan, Italy; marina.camera@cardiologicomonzino.it; 5 Department of Pharmaceutical Sciences, University of Milan, 20133 Milan, Italy; 6 Nephrology and Dialysis Unit, ASST Fatebenefratelli Sacco, 20131 Milan, Italy; gallieni.maurizio@asst-fbf-sacco.it; 7 Department of Biomedical and Clinical Sciences ‘Luigi Sacco’, University of Milan, 20157 Milan, Italy; gidaro.antonio@asst-fbf-sacco.it; 8 Cardiology Unit, IRCCS Orthopedic Institute Galeazzi, 20161 Milan, Italy; luigi.gianturco@asst-rhodense.it; 9 Cardiology Unit, Sant’Anna Hospital, ASST Lariana, 22042 Como, Italy; carlo.piemontese@asst-lariana.it; 10 Division of Cardiology, IRCCS Ospedale San Raffaele, 20132 Milan, Italy; pizzetti.giuseppe@hsr.it; 11 Gastroenterology Unit, Valduce Hospital, 22100 Como, Italy; francoredaelli01@gmail.com; 12 Angelo Bianchi Bonomi Hemophilia and Thrombosis Center, IRCCS Cà Granda Maggiore Hospital Foundation, 20122 Milan, Italy; barbara.scimeca@policlinico.mi.it; 13 Istituto Clinico Città Studi, 20131 Milan, Italy; sebastiano.tadeo@ic-cittastudi.it; 14 School of Medicine and Surgery, University of Milano-Bicocca, 20126 Milan, Italy; giuseppe.bellelli@unimib.it; 15 Acute Geriatrics Unit, San Gerardo Hospital ASST Monza, 20900 Monza, Italy; 16 Department of Cardiology, IRCCS Istituti Clinici Scientifici Maugeri, 20138 Milan, Italy

**Keywords:** atrial fibrillation, DOAC, frailty, old age, cardiovascular prevention, stroke, bleeding

## Abstract

In the last twelve years the clinical management of patients with atrial fibrillation has been revolutionised by the introduction of direct oral anticoagulants. Despite the large amount of evidence produced, some populations remain relatively poorly explored regarding the effectiveness and safety of direct oral anticoagulants, such as the oldest and/or frailest individuals. Frailty is clinical syndrome characterized by a reduction of functions and physiological reserves which results in individuals having higher vulnerability. While current evidence underlines a relationship between atrial fibrillation and frailty, particularly in determining a higher risk of adverse outcomes, data regarding effectiveness and safety of direct oral anticoagulants in frailty atrial fibrillation patients are still lacking, leaving uncertainty about how to guide prescription in this specific subgroup. On these premises, this multidisciplinary consensus document explains why it would be useful to integrate the clinical evaluation performed through comprehensive geriatric assessment to gather further elements to guide prescription of direct oral anticoagulants in such a high-risk group of patients.

## 1. Introduction

In the last twelve years the clinical management of patients with atrial fibrillation (AF) has been revolutionised by the introduction of direct oral anticoagulants (DOACs) for the management of thromboembolic risk [1,2]. Since 2009, a progressive increase in the use of DOACs has been reported [3,4]. Epidemiological data have shown how this change in clinical practice has led to a steady and continuous reduction of stroke risk, with contemporary cohorts showing very low incidence rate for thromboembolic events [5,6]. Despite the large use of DOACs, even beyond the area of AF management, some areas remain unexhaustively covered by evidence. It’s unclear the effectiveness and safety of these drugs in the oldest and/or frailest individuals with AF, and thus whether the DOACs have the same indications in this population as in the general population. Noteworthy, in the most recent European guidelines for AF clinical management, no specific recommendations were released regarding the specific management of these patients [7]. Such a lack of evidence leaves strong uncertainties in clinical practice. This is particularly relevant considering the strong relationship between AF and old age, which plays a significant role regarding both the rise in the incidence and prevalence of AF and the clinical course of patients with AF, conferring a higher risk of adverse outcomes for all [7].

In daily clinical practice, all specialists face increasing complexity, requiring a modern approach that integrates information from large studies and evaluation of the individual patient.

Based on these considerations, a multidisciplinary group of physicians have cooperated to examine the evidence available about this issue, with the aim to reach a consensus on the current knowledge and assess the future needs in terms of research, clinical and educational scenarios.

## 2. Use of DOACs in Atrial Fibrillation

Use of warfarin and other vitamin K antagonist (VKA) anticoagulant drugs has been for a long time the only therapeutic choice to reduce thromboembolic risk in AF patients. Even though VKAs have proved to be effective, the residual bleeding risk, together with a number of limitations, including the narrow therapeutic window to achieve desired anticoagulation, the difficulties in monitoring anticoagulation serum levels and the risk of drugs and food interactions, have always limited uptake of such therapies [8,9]. To overcome the significant limitations of VKAs, in the early 2000s several drugs were developed tested. Four of these molecules reached approval and entered routine clinical practice from 2009. Dabigatran is a direct thrombin inhibitor, while apixaban, rivaroxaban and edoxaban are direct factor Xa inhibitors. All of them have been proved to be non-inferior to warfarin in terms of prevention of stroke, and superior to warfarin regarding the risk of major bleeding occurrence [10,11,12,13]. Furthermore dabigatran and apixaban were slightly superior to warfarin in reducing the risk of both stroke and thromboembolic events [10,12]. All the DOACs share similar characteristics, but also differ from each other in relation to their pharmacodynamic and pharmacokinetic properties. The main features of the four approved DOACs are reported in Table 1. In summary, the prodrug Dabigatran shows the lowest bioavailability, while rivaroxaban has the highest if taken with a hearty meal. Renal clearance was lowest for apixaban and highest for dabigatran. All the factor Xa inhibitors are partly metabolised by CYP3A4, while absorption of edoxaban and rivaroxaban is significantly influenced by food intake.

After approval, data coming from randomized controlled trials (RCTs) showed that all the DOACs, considered as a class, had a significant advantage in reducing the risk of stroke and thromboembolic events when prescribed at their full dose (risk ratio [RR] 0.81, 95% confidence interval [CI] 0.73–0.91) compared to warfarin [15]. Even more importantly, a meta-analysis including the first RCTs on the four DOACs highlighted how the risk of major bleeding was significantly lower (RR 0.86, 95% CI 0.73–1.00) for these drugs compared to warfarin. [15] This effect was particularly driven by the reduction in risk of intracranial hemorrhage (RR 0.48, 95% CI 0.39–0.59) [15]. It also became clear how DOACs significantly reduced the risk of all-cause death (RR 0.90, 95% CI 0.85–0.95) [15]. Following the marketing of the four DOACs, much observational data have been released, substantially confirming RCT effectiveness and safety [6,16]. Notwithstanding, real-life data highlighted how often AF patients are inappropriately prescribed with low-dose DOACs [17], which was found to be associated with an increased risk of adverse outcomes [17,18].

While no direct comparisons exist between the various DOACs, indirect comparisons made through systematic reviews and meta-analyses highlighted some differences among them [19]. In particular, apixaban seemed to have a significantly better safety profile [19,20], with comparable effects on efficacy outcomes. However, a recent network meta-analysis suggests that apixaban should be considered as a first choice regarding the reduction of stroke and thromboembolic events risk [20].

In relation to older patients, data coming from the RCTs, when pooled together, reported slightly different results than the overall cohort of AF patients, with a significant reduction in stroke risk (RR 0.70, 95% CI 0.61–0.80) and no significant differences in major bleeding risk (RR 0.91, 95% CI 0.72–1.16) for patients ≥75 years old [21]. A larger meta-analysis, including data from observational studies, confirmed such results, providing evidence of a lower risk for all-cause death (HR 0.77, 95% CI 0.65–0.92) and intracranial bleeding (HR 0.58, 95% CI 0.50–0.67) in AF patients ≥75 years old [22]. Furthermore, the same systematic review suggested that apixaban seems to have the best effectiveness and safety profile in AF subjects ≥75 years of age, pointing out how scarce evidence is currently available on the differential impact of DOACs compared to VKAs in regards to main geriatric syndromes (multimorbidity, polypharmacy, falling risk, dementia and frailty) [22].

## 3. Definition of Frailty and Impact on Atrial Fibrillation

Frailty is a medical syndrome characterized by a progressive decline in homeostatic and physiological reserves. It exposes the individual to an increased vulnerability to internal and external stressors, leading to an increased risk for adverse outcomes [23,24]. In the last years, evidence has been accumulating that frailty is not exclusively peculiar to older subjects and that high levels of frailty can be found in younger patients affected by diverse specific conditions, including cardiovascular diseases [25,26,27].

Many tools have been developed to assess frailty [28]. Notwithstanding, two main methods are extensively used to describe it: (i) the frailty phenotype, which is substantially based on the evaluation of residual physical strength [29]; (ii) the frailty index (FI), which is based on the concept that frailty is the result of the cumulative juxtaposition of various health deficits, provided that these are biologically determined [30,31]. Irrespective of its definition, frailty is associated with a significantly higher risk of death [32,33] and disability [34], as well as with higher healthcare costs [35].

A relationship between AF and frailty has been reported [36]. Data coming from the available literature reports that prevalence of frailty among AF patients is substantial, being between 1.6% and 56.2% according to the various studies [36]. In a retrospective study by Pilotto et al. [37], frailty was evaluated by a modified version of the multidimensional prognostic index (MPI). This tool includes the assessment of cognitive function, pressure sore risk, autonomy in activities of daily living, mobility, and presence of social support. According to MPI, a quarter of the patients (26.7%) were frail, while 34.7% were prefrail. In the studies by Madhavan [38] and Saczynski [39], which evaluated frailty according to the frailty phenotype, a different prevalence of frailty was reported (5.9% and 13.8%, respectively), reflecting differences in the inclusion criteria. Indeed, in the first one, all patients ≥18 years of age were included, while in the second only those ≥65 years were enrolled.

Four major studies [40,41,42,43] were reported using a FI to assess frailty, adopting the same cumulative deficit model proposed by Rockwood and Mitnitski [30,31]. The FI computed in the four studies considered different number of deficits, retrieving data from different sources (i.e., clinical charts, electronic records, hospital-based electronic dataset) and including patients according to different inclusion criteria and study designs (Table 2). All these differences might likely determine the large variability in prevalence of frail subjects, ranging from 1.6% in the study by Yang and colleagues [42] to 59.1% in the study by Wilkinson and colleagues [40].

Beyond its prevalence, frailty has been reported to significantly impact the prescription of OAC. However, current data are controversial, reporting both studies in which frail patients are significantly less prescribed and studies in which no difference in prescription has been reported [36]. A role seems to be played by the clinical setting in which the patients are seen. Indeed, a previously published systematic review found that if in-hospital frail patients are usually less likely prescribed with OAC, frail AF patients taken from large cohort studies are, conversely, more prescribed than robust ones [44]. Moreover, only few data exist about the role of DOACs compared to VKAs in frail patients [36]. In a secondary analysis derived from the ENGAGE AF-TIMI 48 trial, if a significant benefit on the risk of major bleeding was reported for both edoxaban doses (30 and 60 mg) compared to warfarin in patients with mild-to-moderate frailty, no difference was found for the risk of thromboembolic events, in all the levels of frailty and with any dose of edoxaban [40]. Furthermore, in severely frail patients, no difference was shown for any of the secondary outcomes, except for the composite endpoint including disabling stroke, life-threatening bleeding, and/or death which in patients randomised to edoxaban 60 mg resulted in a reduction [40] (HR = 0.66, 95% CI = 0.39–0.99). In the study by Martinez et al., with the exception of a lower rate of stroke and systemic embolism in rivaroxaban users compared to VKAs users, no significant difference was reported among AF frail patients using DOACs [45]. Conversely, in the study by Lip and colleagues, derived from the large ARISTOPHANES observational registry, in frail AF patients, apixaban was found to be associated both with a lower risk of stroke and major bleeding, while rivaroxaban was associated with a higher risk of major bleeding than VKAs [46].

Even regarding the impact of frailty on outcomes in patients with AF, there is no substantial clarity [36]. While a significant higher risk of all-cause death was found in all the studies investigating this issue [36], it is still unclear if, and to what extent, frailty would interact with risk of stroke and major bleeding [36]. In the analysis performed on the ORBIT-AF registry, while frailty was associated with an increased risk for all the outcomes examined in the unadjusted analysis, after multiple adjustments only the relationship with all-cause death (HR = 1.29, 95% CI = 1.08–1.55) remained statistically significant [38]. Wilkinson and colleagues reported a significant association between levels of frailty only with risk of all-cause death and gastrointestinal bleeding [41]. In other analyses derived from RCTs and observational studies, a positive association between frailty and risk was found for all the outcomes related to AF [40,43].

Very recently, more evidence has emerged about the effectiveness and safety of DOACs in frail patients. In a propensity-matched analysis derived from Medicare, according to the burden of frailty as measured with a claims-based FI, effectiveness and safety of dabigatran, rivaroxaban and apixaban compared to warfarin were evaluated. Use of dabigatran showed similar effectiveness and safety in robust, prefrail and frail patients; rivaroxaban use indicated a small reduction in risk of stroke but a higher risk of major bleeding in frail patients, while use of apixaban revealed a substantial and consistent reduction of all major adverse outcomes in frail patients compared to warfarin [47]. Larger evidence is still lacking about the other DOACs.

## 4. Guidelines Recommendations about Older and Frail Adults

Generally speaking, clinical practice guidelines lack specific recommendations regarding the management of older and frail adults with AF [2]. For instance, in the 2020 ESC version of the AF guidelines, only a small paragraph reports on frail people, simply stating that OAC is indicated and DOACs appear to have better risk-benefit profile than VKAs. However, frailty is not specifically defined and substantially and erroneously overlaps with the idea of ‘older age’ [7].

Nonetheless, in the very recent European Heart Rhythm Association 2021 practical guide on the use of DOACs, the experts were more accurate in defining the concept of frailty and proposed the Clinical Frailty Scale to operationalise frailty [48]. Even though they still avoided the release of specific recommendations, it is clearly stated that frailty should not be considered as a reason to withhold OAC, though it is recognized that frailty can influence the risk of adverse outcomes, and in particular the bleeding risk [48]. Additionally, they generally discuss the idea that in severely frail patients, use of OAC may not be beneficial, although this statement is not clearly supported by scientific evidence [48].

## 5. Synthesis and Proposal for Clinical Assessment

By examining the evidence in the current literature, it emerges that if frailty is a significant clinical issue in the management of AF patients, it is still unclear on which extent it influences the choice of DOACs vs. VKAs and whether the use of DOACs is beneficial in patients with higher frailty levels. Even AF experts still do not have enough confidence with this issue to release specific and straightforward recommendations regarding this specific cohort of patients.

In the context of AF, it is nowadays accepted and recommended that patients with frailty should be looked after more comprehensively and beyond the mere balance between thromboembolic and bleeding risk [7]. Indeed, epidemiological evidence highlights that the risk of death, both from cardiovascular and non-cardiovascular causes, represents an urgent and pivotal clinical problem [49,50]. In order to face this issue, the 2020 ESC guidelines on AF management recommend an integrated holistic approach to consider the larger set of concomitant risk factors and comorbidities affecting the survival of AF patients [7]. In particular, the ESC guidelines recommend the ‘Atrial Fibrillation Better Care’ (ABC) pathway to streamline the application of integrated management in AF patients [7]. Actually, this approach has been found to be significantly associated with a consistent reduction of risk for all the major AF-related adverse clinical outcomes [51].

From confronting the ideas and opinions of our large set of competences, thanks to the multidisciplinary composition (cardiology, geriatrics, gastroenterology, nephrology, pharmacology, public health, internal medicine), this working group believes that such an approach, considering all the clinical issues specific to a frail AF patient, could be useful to tailor the prescription of the most appropriate OAC drug for each patient. This approach would also represent the best way to pay attention to all the risks connected with AF, which is also known to be dynamic and changeable over time [52].

Based on the idea that a comprehensive evaluation of the patient is the best way to characterize the risk of adverse outcomes, we do believe that the geriatric comprehensive assessment (GCA) could be placed aside to an integrated approach, such as the ABC pathway. Use of GCA has been suggested as an effective way to manage frailty in older adults [53]. The conjunction between an integrated approach to AF patients’ management and GCA could probably provide an adequate way to evaluate, characterize and stratify risk in AF frail patients. Carefully evaluating the residual physiological functions and planning specific interventions could help to reduce the impact of frailty on the AF-related outcomes. Moreover, taking adequate consideration of all clinical characteristics and physiological reserves could aid the physicians to choose the right OAC drug, either a DOAC or VKA, minimize the risk of adverse events and optimize the reduction of thromboembolic and death events. While this proposal for action appears reasonable based on current evidence, it is clearly lacking adequate support from experimental data. Our initiative provides an overall assessment of the current epidemiological and clinical knowledge, strongly emphasizing the need for further research aimed at examining whether a similar approach would be beneficial, with the aim of choosing the best treatment strategies and minimizing the impact of frailty.

Regarding the choice between VKAs and DOACs, while the effectiveness and safety of DOACs in the general AF population is undeniable, data are still substantially lacking about specific geriatric patients affected by several of the most widespread geriatric syndromes, as underlined in the work by Grymonprez and colleagues (Table 2) [22], although the available data suggest that treating frail patients affected by AF with apixaban could guarantee significantly better efficacy and safety than warfarin, also because of relatively greater availability of data relating to geriatric subgroups (Table 2) [22,47]. Indeed, as Table 2 underlines, while data on the role of apixaban in patients with dementia and polypharmacy are still limited, there are more data on other subgroups (such as very old patients, those with high risk of falling, and with multimorbidity), which are lacking for other DOACs [22,47].

In this context the specific role of impaired renal function should also be taken in mind, which is often prevalent in older individuals, and can significantly influence the management of DOACs [54,55,56], with apixaban appearing the more effective and safer choice [57,58]. Nevertheless, we do recommend some caution in prescribing DOACs for those AF patients reporting significant geriatric conditions (Table 2). This is also in line with some recent data stressing the need for a new framework to rethink and re-evaluate the use of OAC in patients with limited life expectancy due to extremely complex clinical situations [59,60]. Cardiologists also agree in recommending caution in prescribing OAC based on the burden of frailty [48].

In conclusion, in AF patients the presence of frailty significantly influences clinical management, even though there is still little evidence about the best approach in prescribing OACs. It is still unclear how to guide the prescription of DOACs and the actual benefit in this specific clinical scenario. We propose, in addition to an integrated clinical approach taking in proper account all the clinical aspects related to AF, the use of GCA, which could provide further evaluation and characterization of these patients, guiding the choice of the right OAC drug for the right patient. This would help to manage frailty and minimize its impact on the natural history of AF patients.

## Figures and Tables

**Table 1:** Comparison between DOACs main characteristics (Based on [14]).

	Dabigatran	Apixaban	Edoxaban	Rivaroxaban
**Oral Bioavailability**	3–7%	50%	62%	80–100% w/food (66% w/out food)
**Renal Clearance**	80%	27%	50%	35%
**Plasma Protein Binding**	35%	87%	55%	95%
**Dialysability**	50–60% (partly dialysable)	14% (partly dialysable)	NA (partly dialysable)	NA (partly dialysable)
**CYP3A4 Metabolism**	No	Yes (Moderate)	Minimal	Yes
**Food Effect on Absorption**	No effect	No effect	6–22% more; minimal effect on exposure	39% more (see above)
**H2 B/PPI on Absorption**	12% to 30% less (not clinically relevant)	No Effect	No Effect	No Effect
**Effect of Asian ethnicity**	+25%	No effect	No Effect	No Effect
**Half-life**	12–17 h	12 h	10–14 h	5–9 h (young) 11–13 h (elderly)
**Other relevant notes**	Dyspepsia (5–10%)	-	-	Intake with food is mandatory

H2 = Histamine H2 Receptor; B = Blockers; PPI = Proton Pump Inhibitor; NA = Not Available.

**Table 2:** Geriatric Conditions with Limited Evidence on DOACs.

**(i) Dabigatran**	
*Effectiveness*	*Safety*
Polypharmacy, High Falling Risk, Frailty, Dementia	Multimorbidity, Polypharmacy, High Falling Risk, Dementia
**(ii) Rivaroxaban**	
*Effectiveness*	*Safety*
Multimorbidity, High Falling Risk, Dementia	Multimorbidity, Polypharmacy, High Falling Risk, Frailty, Dementia
**(iii) Apixaban**	
*Effectiveness*	*Safety*
Polypharmacy, Dementia	Polypharmacy, Dementia
**(iv) Edoxaban**	
*Effectiveness*	*Safety*
Older Age, Multimorbidity, Polypharmacy, High Falling Risk, Frailty, Dementia	Older Age, Multimorbidity, Polypharmacy, High Falling Risk, Frailty, Dementia
